# Supplementary material for: A framework for the risk prediction of avian influenza occurrence: An Indonesian case study
Source: PLoS One. 2021 Jan 15;16(1):e0245116. doi: 10.1371/journal.pone.0245116 (PMC7810353; doi:10.1371/journal.pone.0245116)
Supplement: S1 File — (ZIP) [file pone.0245116.s001.zip › supplementary.pdf]

A Framework for Risk Assessment of Avian Influenza Occurrence: An Indonesian Case Study

Samira Yousefinaghani[§], Rozita Dara[§], Zvonimir Poljak[†], Fei Song[§], and Shayan Sharif[ς]

[§]School of Computer Science, University of Guelph, Guelph, Ontario, Canada

[†]Department of Population Medicine, Ontario Veterinary College, University of Guelph, Guelph, Ontario, Canada

[ς]Department of Pathobiology, University of Guelph, Guelph, Ontario, Canada

December 18, 2020

Table 1: Table S1. Description of fields in Fig 2. Tables indonesia_testset and indonesia_training

**Attribute**	**Type**	**Description**	**Range**
year	YEAR	the year that the event occurred in	[2009 2017]
week	INT(11)	the week number that the event occurred in	[1 52]
start_day_of_week	DATE	the day number that the event occurred	
cell_num	INT(11)	the cell number	[63 956]
cell_longitude	VARCHAR(50)	the longitude of the cell centre	[92.764 141.021]
cell_latitude	VARCHAR(50)	the latitude of the cell centre	[-11.606 6.519]
event_ids	VARCHAR(200)	the identification number of events that have occurred in the defined scale	[3,830 233,202]
density_Ch	DOUBLE	the density of chickens	[15.43 926,952.04]
density_Dk	DOUBLE	the density of ducks	[0.93 44,751.55]
temperature	DOUBLE	the temperature of the centre of cell	[32.34 95.51]
humidity	DOUBLE	the humidity of the centre of cell	[0.23 1]
windSpeed	DOUBLE	the wind speed of the centre of cell	[0 104.69]
pressure	DOUBLE	the pressure of the centre of cell	[945.34 1,033.23]
precipitation	DOUBLE	the precipitation of the centre of cell	[3.92 552.25]
num_cases	INT(11)	the total number of cases of all events in the defined scale	[0 154,000]
bird_existence	INT(11)	weather wild birds are distributed within the cell	0,1
bird_obtained	INT(11)	a control field for obtaining the bird distribution data	0,1

has_event	INT(11)	weather an event occurred in the defined scale	0,1
num_events	INT(11)	the number of events that have occurred in the defined scale	[0 16]
weather_obtained	INT(11)	a controlling field for obtaining the weather data	0,1

Table 2: Table S2. Description of fields in Fig 2.Table events-indonesia

**Attribute**	**Type**	**Description**
id	BIGINT(40)	a unique ID specified for each event
source	VARCHAR(200)	the source of information including, WHO, OIE, National authorities, FAO field officer or media
longitude	VARCHAR(50)	the longitude of the point the event occurred in
latitude	VARCHAR(50)	the latitude of the point the event occurred in
region	VARCHAR(200)	the continent of the event
location	VARCHAR(200)	the country of the event
location_id	INT(10)	the ID of the location of event
admin1	VARCHAR(200)	the province name in Indonesian language
state1	VARCHAR(200)	the province name in English language
observationDate	Date	the date in which the event was observed
reportingDate	Date	the date in which the event was reported
status	VARCHAR(200)	whether the event is confirmed or denied
disease	VARCHAR(200)	the name of disease that caused the event
serotypes	VARCHAR(200)	the virus subtype of disease that caused the event
speciesDescription	VARCHAR(200)	name of the species that were involved
sumAtRisk	VARCHAR(200)	the total number of birds at risk
sumCases	VARCHAR(200)	the total number of reported cases
sumDeaths	VARCHAR(200)	the total number of deaths reported
sumDestroyed	VARCHAR(200)	the total number of destroyed reported
sumSlaughtered	VARCHAR(200)	the total number of slaughtered reported
humansGenderDesc	VARCHAR(200)	the gender of humans involved
humansAge	VARCHAR(200)	the age of humans involved
humansAffected	VARCHAR(200)	whether any human was affected by the event
humansDeaths	VARCHAR(200)	whether any human died by the event

Table 3: Table S3. Description of fields in Fig 2. Table waterfowls

Attribute	Type	Description
id	INT(10)	the year in which the distribution has been reported
species_id	VARCHAR(50)	each species has a unique ID
species_name	VARCHAR(50)	Scientific name of the species
origin	ENUM	describes whether that species occurs in an area naturally. It accepts several values including Native,Reintroduced,Introduced,Vagrant,Origin Uncertain and Assisted Colonisation
seasonality	ENUM	describes the the period in the species life cycle in which it occurs in an area. It accepts several fields including Resident,Breeding Season,Non-breeding Season,Passage and Seasonal Occurrence Uncertain
presence	ENUM	describes the level of certainty we have as to the species existence in an area. It accepts several fields including Extant,Probably Extant,Possibly Extant,Possibly Extinct,Extinct and Presence Uncertain
date	YEAR	the year that the the distribution is reported
shape_length	FLOAT	the length of the polygon's perimeter.
shape_area	FLOAT	the area of the polygon's perimeter.
date	YEAR	Year in which the polygon was mapped, compiled or modified
polygon_simple	LONGTEXT	a list of longitude-latitude pairs

Table 4: Table S4. Description of fields in Table indonesia_cells

**Attribute**	**Type**	**Description**
cell_num	INT(11)	the number of cell in the grid
cell_latitude	VARCHAR(50)	the latitude of the centre of cell
cell_longitude	VARCHAR(50)	the longitude of the centre of cell
province	VARCHAR(200)	the name of province in Indonesian language
state1	VARCHAR(200)	the name of province in English language
