# Supplementary material for: A framework for the risk prediction of avian influenza occurrence: An Indonesian case study
Source: PLoS One. 2021 Jan 15;16(1):e0245116. doi: 10.1371/journal.pone.0245116 (PMC7810353; doi:10.1371/journal.pone.0245116)
Supplement: Attachment — Submitted filename: Rebuttal_R1.docx [file pone.0245116.s003.docx]

**Date:** 17 Dec 2020

**Title:** A Framework for Risk Assessment of Avian Influenza Occurrence: An Indonesian Case Study

**Authors:** Samira YousefiNaghani, Rozita Dara, Zvonimir Poljak, Fei Song, and Shayan Sharif

**Manuscript No:** PONE-D-20-03935R1

Dear Dr. Alessandro Rizzo

We are very grateful to Academic Editor and the reviewers for the second review and comments regarding the Manuscript ID PONE-D-20-03935R1 entitled "A Framework for Risk Assessment of Avian Influenza Occurrence: An Indonesian Case Study".

We have addressed the comments and made the required changes which we believe have improved the manuscript in a way that is now acceptable for publication in PLOS ONE journal. In the following, we will describe our responses to the points made by academic editor and reviewers. Given comments have been copied and are followed by specific responses in green.

**Academic Editor Comments:**

- One of the two reviewers is satisfied with your revision. Another reviewer, on the other hand, deems the technical content as adequate, but requires you to clarify some aspects with respect to data treatment. I concur with the reviewer that such clarifications will contribute to make the paper more readable and to significantly increase its visibility and impact.

**Authors:**

- We added a paragraph in the Methodology section to add more clarity on data aggregation. The paragraph is highlighted in the manuscript. For the purpose of reusability, we can provide the data to be publicly available for readers.
- While revising your submission, please upload your figure files to the Preflight Analysis and Conversion Engine (PACE) digital diagnostic tool, <https://pacev2.apexcovantage.com/>. PACE helps ensure that figures meet PLOS requirements. To use PACE, you must first register as a user. Registration is free. Then, login and navigate to the UPLOAD tab, where you will find detailed instructions on how to use the tool. If you encounter any issues or have any questions when using PACE, please email PLOS at [figures@plos.org](mailto:figures@plos.org). Please note that Supporting Information files do not need this step.

**Authors:**

- We uploaded the EPS figures on PACE tool. These figures have been adjusted and generated TIFFs figures. We will be submitting TIFF figures along with the revised manuscript.
- In addition, we renamed the figures to their associated caption names.

**Reviewer #1 comments:**

- Even if authors answered to revision issues, my personal problem with this work is still related to the partially addressed evidence and focus on geographic data aspects. The answer to revision is indeed not so clear with respect to the reviewer invitation on GIS related aspects (i.e. lat and long information at the first revision step). Spatial modules and images are strictly related to epidemiology performance and analysis. Thus, even if authors addressed the reviewer comments they did not mentioned the technical aspects related to mapping gathered data in a native spatial system. Now, if this is not an issue that should be deeply treated, it is necessary in case of: (i) integration of different sources and (ii) in case of reuse of the proposed method for different cases. How data has been mapped into commonly used and structured geographic model, is still not clear. Performing interesting queries as well as epidemiological analysis is what reader should expect from this paper starting from examples, title and abstract. On the other hand, if the proposal is finalized as a presentation of a use case my suggestion is to improve the data structure representation as minor revision to allow readers in gathering the contribution as a well structured example. I thus suggest to review the methodology to enrich possibility of replicating on different data samples.

**Authors:**

- Thanks for your helpful suggestion. On page 6, in the Methodology Section and after Fig 3, we added a paragraph explaining how the relational dataset was used as a geographical model which can be visualized in a GIS system. We added the paragraph below:

*``The data aggregation was performed in a Structured Query Language table. The response variable describes a disease event that occurred within a certain time interval (week number t) and a spatial compartment with a center of (x,y), where x represents longitude and y represents latitude. The spatiotemporal information in ``Indonesia" data tables in Fig 2 are demonstrated by ``week", ``cell_longitude" and ``cell_latitude" attributes. Similarly, for other covariates, pre-processing techniques were applied to transform the records into the defined grid-based table. Since the table included coordinate information for each record, a weekly timeline of information could be visualized in maps using GIS software. Moreover, extracted patterns from the grid-based data could be simple and easy to understand.”*
